# Identification of *SPOP* related metabolic pathways in prostate cancer

**DOI:** 10.18632/oncotarget.21460

**Published:** 2017-10-03

**Authors:** Min Yan, Huan Qi, Jia Li, Guozhu Ye, Yaping Shao, Tongming Li, Jing Liu, Hai-Long Piao, Guowang Xu

**Affiliations:** ^1^ Scientific Research Center for Translational Medicine, Dalian Institute of Chemical Physics, Chinese Academy of Sciences, Dalian 116023, China; ^2^ CAS Key Laboratory of Separation Science for Analytical Chemistry, Dalian Institute of Chemical Physics, Chinese Academy of Sciences, Dalian 116023, China; ^3^ University of Chinese Academy of Sciences, Beijing 100049, China

**Keywords:** SPOP, metabolism

## Abstract

Speckle-type POZ protein (SPOP), as a cullin-based E3 ubiquitin ligase, has been identified as one of the most frequently mutated genes in prostate cancer (PCa). However, whether *SPOP* mutations contribute to metabolic reprogramming in PCa remains unknown. Here, integrated studies of transcriptomics and metabolomics as well as lipidomics were performed in matched PCa tumor (PCT) and adjacent non-tumor (ANT) tissues, followed by correlation analysis of *SPOP* mutations with altered metabolic pathways in *SPOP*-mutated PCa patients. Interestingly, transcriptomics profiling showed that all *SPOP* mutations (with 16.7% frequency, 11/66) occurred at the conserved residues in the substrate binding domain of meprin and TRAF homology (MATH). The results of integrated analysis indicated that three metabolic pathways, including tricarboxylic acid (TCA) cycle, fatty acid metabolism and glycerophospholipid metabolism, exhibited obvious upregulation in *SPOP*-mutated PCT tissues. Furthermore, both correlation analyses based on integrated data and cBioportal revealed that *FH, ELOVL2* and *ACADL* genes might be involved in *SPOP*-mutation-related upregulation of these metabolic pathways. Taken together, our study provided new insights in understanding the relationship between metabolic pathways and *SPOP* mutations in PCa.

## INTRODUCTION

Prostate cancer (PCa) is the second most common cancer and the fifth leading cause of death from malignant carcinoma in men [1, 2]. It is estimated that approximately 603,000 men were diagnosed with PCa and 266,000 men died of PCa in China in 2015 [3]. The incidence rates of PCa have shown an increasing trend in almost all countries [4]. However, the pathogenesis and etiology of PCa remain largely unknown. *SPOP* has been identified as one of the most frequently mutated genes in PCa with 6–15% mutation rate [5]. SPOP is a cullin-based E3 ubiquitin ligase, involving meprin and TRAF homology (MATH) protein interaction domain and Bric-a-brac/Tramtrack/Broad complex (BTB) domain [6]. Most of *SPOP* mutations have been reported to occur in conserved residues at MATH domain which play key roles in substrate interaction [5, 6]. Previous studies demonstrated that the ubiquitination complex of SPOP-CUL3 can regulate the degradation of various substrates, such as DEK proto-oncogene (DEK) [7], nuclear receptor coactivator 3 (NCOA3) [8], androgen receptor (AR) [9], V-Ets avian erythroblastosis virus E26 oncogene homolog (ERG) [10, 11] and cell division cycle 20 (CDC20) [12]. Thereby, the function of degrading oncogenic substrates of SPOP can be abrogated by *SPOP* mutations, which can partly explain the reason for PCa initiation and progression.

Accumulating evidence supports the notion that metabolic pathway reprogramming plays a critical role during cancer progression in various types of neoplasias including PCa [13–16]. One of the most well-known metabolic characteristics observed in tumor cells is the Warburg effect, in which ATP and lactate are produced by high rates of glycolysis instead of oxidative phosphorylation [17]. Several recent studies have revealed the occurrence of metabolic reprogramming in PCa. Jonathan *et al.* demonstrated that almost thirty metabolites exhibited statistically significant changes in aggressive prostate tumors relative to cancer-free prostate tissues, including amino acid catabolites, lipid compounds and energetics-related metabolites [18]. Metabolomics profiles in PCa clinical samples showed that the accumulation of sarcosine, a methylated metabolite of the amino acid glycine, positively correlates with PCa progression [19]. Also, previous findings from our laboratory identified sphingosine and cholesteryl oleate as potential molecular biomarkers to distinguish PCa and benign prostatic hyperplasia [20, 21]. Importantly, a recent review by Liu *et al.* reported that fatty acids oxidation is a main pathway for producing energy in PCa [22]. Furthermore, various proteins have been determined to be involved in metabolic pathway reprogramming in neoplastic prostate cells, such as AR [23], steroid receptor coactivator 2 (SRC-2) [24], MYC and AKT [25]. However, whether *SPOP* mutations are associated with metabolic reprogramming in PCa has not been explored before.

In this study, transcriptomics profiling was applied for 66 matched prostate cancer tumor (PCT) and adjacent non-tumor (ANT) samples. Mutation information of corresponding patients was obtained. Additionally, to obtain the comprehensive landscapes of metabolic alterations in PCa patients, metabolomics and lipidomics were performed by gas chromatography-mass spectrometry (GC-MS) and liquid chromatography-mass spectrometry (LC-MS) in matched PCT and ANT tissues. Moreover, we characterized the metabolites and metabolic enzymes related to *SPOP* mutations by integration of transcriptomics and metabolomics as well as lipidomics.

## RESULTS

### Patient information and genetic alterations in PCa patients

**Figure 1:**
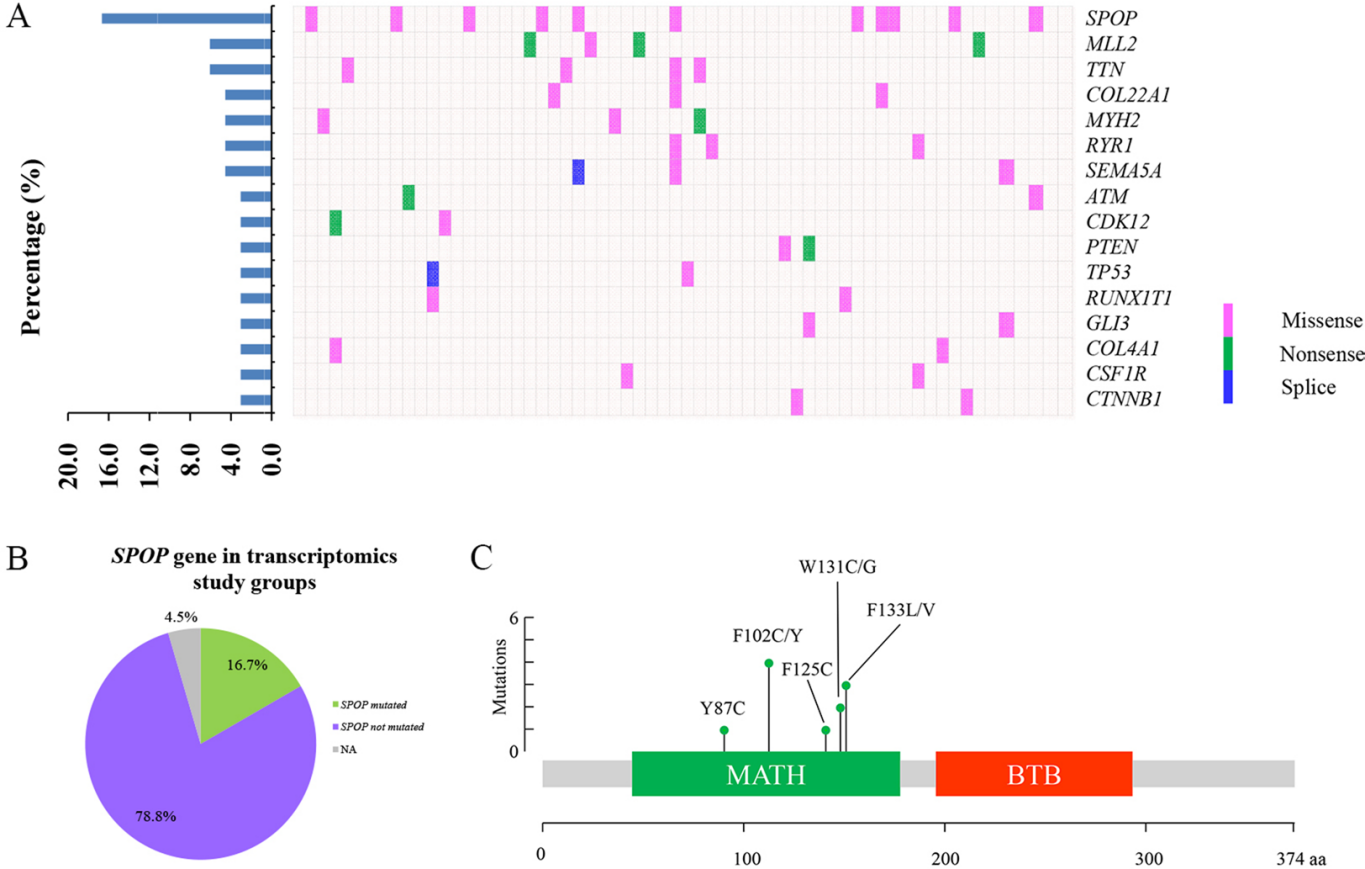
The frequently mutated genes in PCa patients (**A**) The percentages of mutated genes in prostate cancer patients (left). The distribution map of mutated genes in PCa patients, each column represents a patient and each row represents a mutated gene (right). (**B**) The distribution map of *SPOP* mutations in transcriptomics study cohort. (**C**) The position of somatic alterations in *SPOP* across the 11 *SPOP*-mutated PCa cohorts.

In this study, 66 patients with PCa were enrolled for transcriptomic analysis and characterized by the clinical pathological features (Table 1). The expression data of 66 matched PCT and ANT tissues were profiled at transcriptional level by RNA-Seq analysis. As shown in Figure 1A, 16 highly mutated genes were found in PCT tissues compared with matched ANT tissues, including *SPOP*, myeloid/lymphoid or mixed-lineage leukemia 2 (*MLL2*), titin (*TTN*), collagen type XXII alpha 1 (*COL22A1*), myosin heavy chain 2 (*MYH2*), ryanodine receptor 1 (*RYR1*), semaphorin 5A (*SEMA5A*), ATM serine/threonine kinase (*ATM*), cyclin dependent kinase 12 (*CDK12*), phosphatase and tensin homolog *(PTEN*), tumor protein P53 (*TP53*), RUNX1 translocation partner 1 (*RUNX1T1*), GLI family zinc finger 3 (*GLI3*), collagen type IV alpha 1 chain (*COL4A1*), colony stimulating factor 1 receptor (*CSF1R*), and catenin beta 1 (*CTNNB1*). Among these mutated genes, *SPOP* was identified as the most frequently mutated one in PCT tissues with a 16.7% frequency, which was consistent with the previous report [5] (Figure 1A–1B). It was interesting that all *SPOP* mutations occurred at the conserved residues in the substrate-binding cleft of MATH (Figure 1C, Table 2).

**Table 1:** Clinical characteristics of PCa patients used for transcriptomics analysis

Characteristics		Total number	Percentage (%)
Gleason score	3 + 3	5	7.6
	3 + 4	23	34.9
	3 + 5	1	1.5
	4 + 3	13	19.7
	4 + 4	10	15.2
	4 + 5	5	7.6
	5 + 4	7	10.6
	5 + 5	2	3.0
Pathological stage	T2cN0M0	36	54.6
	T2cN1M0	2	3.0
	T3aN0M0	9	13.6
	T3aN1M0	1	1.5
	T3bN0M0	9	13.6
	T3bN1M0	2	3.0
	T4N0M0	5	7.6
	NA	2	3.0
Pathological progression	Localized	36	54.6
	Locally advanced	23	34.9
	Metastatic	6	9.1
	NA	1	1.5

NA indicated that the information is missing.

**Table 2:** Mutation information and clinical characteristic of 11 *SPOP*-mutated PCa patients.

Sample	Pathological progress	Gleason score	Function	Amino acid change
*SPOP*_M Patient 1^*^	localized	3 + 3	missense	p.W131G
*SPOP*_M Patient 2	locally advanced	3 + 4	missense	p.F125C
*SPOP*_M Patient 3^*^	localized	3 + 4	missense	p.Y87C
*SPOP*_M Patient 4	locally advanced	4 + 3	missense	p.F102C
*SPOP*_M Patient 5	locally advanced	4 + 3	missense	p.F102C
*SPOP*_M Patient 6^*^	localized	4 + 3	missense	p.F133V
*SPOP*_M Patient 7	locally advanced	4 + 4	missense	p.F133V
*SPOP*_M Patient 8	localized	4 + 5	missense	p.W131C
*SPOP*_M Patient 9^*^	localized	4 + 5	missense	p.F133L
*SPOP*_M Patient 10	locally advanced	5 + 4	missense	p.F102C
*SPOP*_M Patient 11^*^	locally advanced	5 + 5	missense	p.F102Y

^*^ Samples were used in metabolomics and lipidomics analysis.

### Differential metabolites between the matched PCT and ANT tissues in *SPOP*-mutated PCa patients

**Figure 2:**
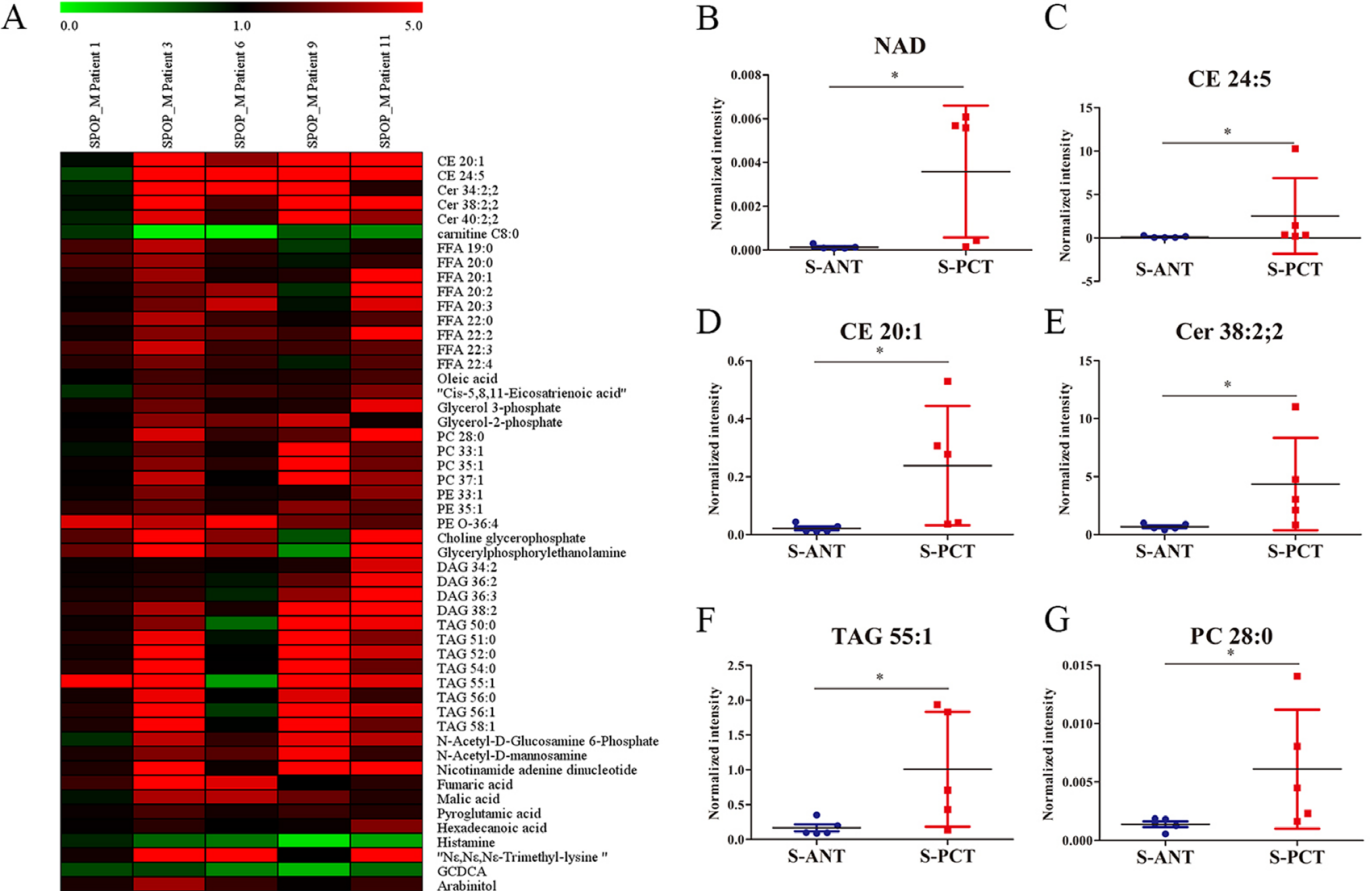
Differential metabolites between the matched PCT and ANT tissues in *SPOP*-mutated PCa patients (**A**) Heatmap of 51 differential metabolites between the matched PCT and ANT tissues in *SPOP*-mutated cohort. (**B–G**) The normalized peak intensity values of the top six remarkably upregulated metabolites including NAD (B), CE 24:5 (**C**), CE 20:1 (**D**), Cer 38:2,2 (**E**), TAG 55:1 (**F**), PC 28:0 (G) are shown. ^*^p < 0.05 compared with the ANT tissues. S-PCT, *SPOP*-mutated PCT tissues; S-ANT, *SPOP*-mutated ANT tissues.

Although *SPOP* is the most frequently mutated gene in PCa, the metabolic pathways regulated by *SPOP* are still unclear. To uncover the potential *SPOP*-related metabolic pathways, we profiled metabolite alterations using GC-MS and LC-MS in *SPOP*-mutated PCT tissues and their matched ANT tissues. A total of 51 differential metabolites were identified in *SPOP*-mutated PCT tissues relative to the matched ANT tissues (Supplementary Table 1). Of these, only 3 metabolites including glycochenodeoxycholic acid (GCDCA), histamine and carnitine C8:0, significantly decreased in *SPOP*-mutated PCT tissues, whereas other 48 metabolites (lipids, organic acids, carbohydrates etc.) increased obviously (Supplementary Table 1, Figure 2A). The top six remarkably upregulated metabolites were nicotinamide adenine dinucleotide (NAD), cholesteryl ester (CE) 24:5, CE 20:1, ceramide (Cer) 38:2, 2, triacylglycerol (TAG) 55:1 and phosphatidylcholine (PC) 28:0 (Figure 2B–2G). These results suggest that several metabolic pathways may be associated with *SPOP* mutations.

### Integration of transcriptomics, metabolomics and lipidomics in *SPOP*-mutated PCa patients

One omics was insufficient to illustrate the alterations occurring in PCa, hence integrated studies of transcriptomics, metabolomics and lipidomics data were performed to further identify the metabolic pathways related to *SPOP* mutations. Gene set analysis was applied for finding altered metabolic pathways in 11 *SPOP*-mutated patients using Kyoto Encyclopedia of Genes and Genomes (KEGG) database [26] by online software Consensuspathdb [27]. Total 3766 differential transcripts were used for gene set analysis to calculate the related metabolic pathways, of which 1840 transcripts were upregulated whereas 1926 transcripts were down-regulated. Most of the related metabolic pathways were significantly upregulated in PCT tissues of *SPOP*-mutated cohort (Table 3). Only two metabolic pathways involving thyroid hormone synthesis and arachidonic acid metabolism were down-regulated in *SPOP*-mutated PCT tissues.

**Table 3:** Gene set analysis of 11 *SPOP*-mutated PCa patients was performed by KEGG database

Pathway name	Candidates contained
Thyroid hormone synthesis^*^	19
Valine, leucine and isoleucine degradation	14
Oxidative phosphorylation	26
Cysteine and methionine metabolism	10
Pyrimidine metabolism	19
Glycine, serine and threonine metabolism	10
Alanine, aspartate and glutamate metabolism	9
Arachidonic acid metabolism^*^	12
Fatty acid degradation	10
Purine metabolism	26
Fructose and mannose metabolism	8
Pyruvate metabolism	9
Butanoate metabolism	7

^*^Down-regulated metabolic pathways in *SPOP* mutated PCT tissues. Others were upregulated metabolic pathways (non-labeled).

The results were calculated by consensuspathdb online software. 3766 differential transcripts were used for gene set analysis, of which 1840 transcripts were upregulated and 1926 transcripts were down-regulated. Candidates contained indicates the number of altered transcripts involved in these metabolic pathways in our study. The altered metabolic pathways with *p* < 0.01 are listed.

Next, to understand *SPOP*-regulated metabolic pathways, we combined the transcriptomics data with our metabolomics and lipidomics data from *SPOP*-mutated cohort and constructed an overall network of metabolic pathways. As presented in Supplementary Figure 1, four sections of metabolic pathways including glycolysis, TCA cycle, fatty acid metabolism and glycerophospholipid metabolism, exhibited obvious alterations in *SPOP*-mutated PCT tissues. Moreover, most of differential metabolites involved in these metabolic pathways were significantly upregulated in *SPOP*-mutated PCT tissues, which were consistent with the altered trends of the relevant transcripts.

**Figure 3:**
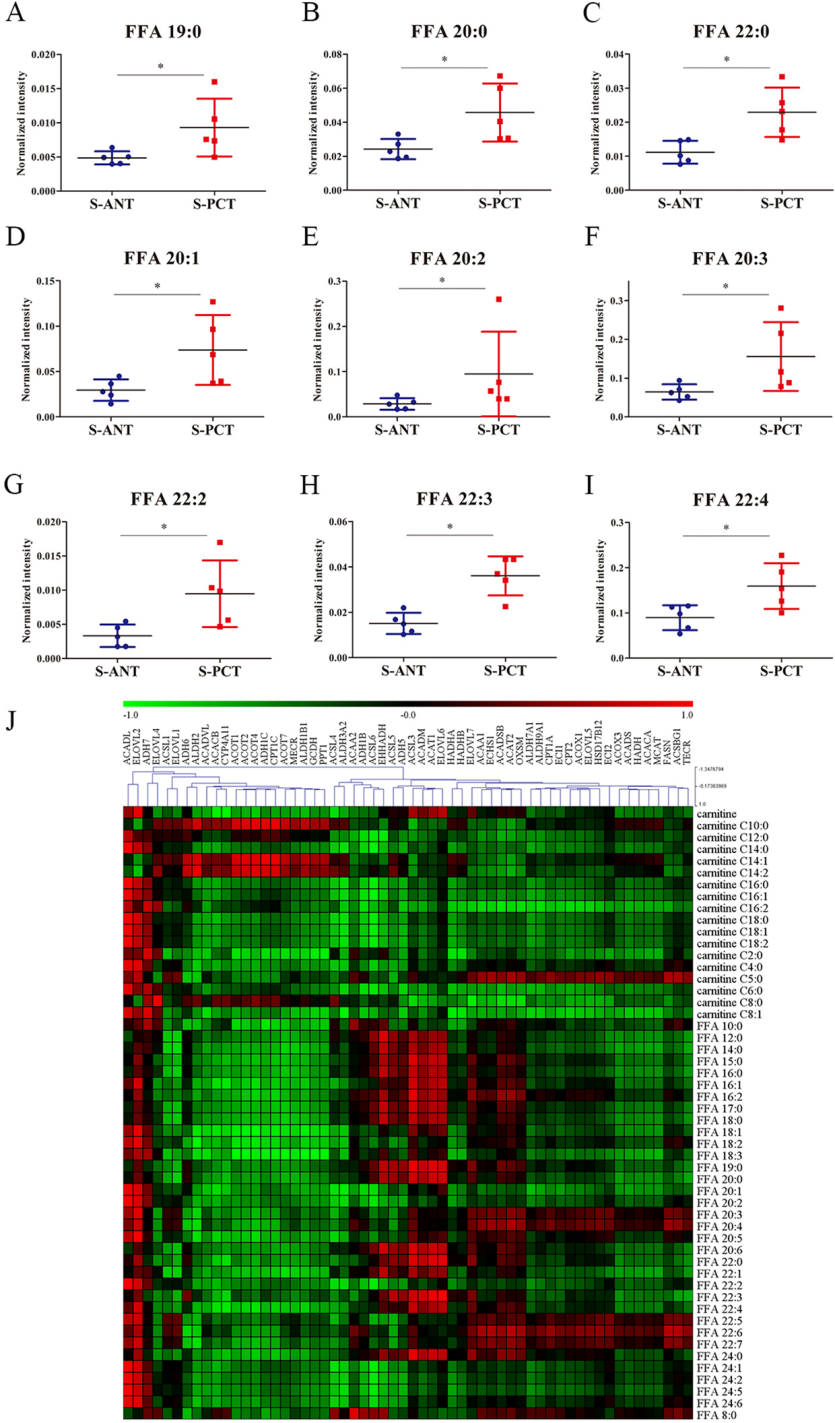
The alterations in fatty acid metabolic pathway in SPOP-mutated cohort (**A**–**I**) The normalized peak intensity values of FFA 19:0 (A), FFA 20:0 (B), FFA 22:0 (C), FFA 20:1 (D), FFA 20:2 (E), FFA 20:3 (F), FFA 22:2 (G), FFA 22:3 (H), FFA 22:4 (I) are shown. (**J**) Heatmap of PCC between 58 transcripts and 52 metabolites in fatty acid metabolic pathway. Each column represented a transcripts and each row represented a metabolites. ^*^p < 0.05 compared with the ANT tissues. S-PCT, *SPOP*-mutated PCT tissues; S-ANT, *SPOP*-mutated ANT tissues.

Increasing evidence indicates that altered fatty acid metabolism, including biosynthesis, transportation and degradation, frequently occurs during PCa progression [22, 28]. Our transcriptomics data showed that 5 genes involved in fatty acid biosynthesis such as fatty acid synthase (*FASN*), acetyl-CoA carboxylase alpha (*ACACA*), malonyl-CoA-acyl carrier protein transacylase (*MCAT*), ELOVL fatty acid elongase 2 (*ELOVL2*) and 3-oxoacyl-ACP synthase, mitochondrial (*OXSM*), 6 genes involved in fatty acid oxidation including acyl-CoA dehydrogenase, long chain (*ACADL*), hydroxyacyl-CoA dehydrogenase/3-ketoacyl-CoA thiolase/enoyl-CoA hydratase (trifunctional protein), beta subunit (*HADHB*), acyl-CoA dehydrogenase, short/branched chain (*ACADSB*), enoyl-CoA hydratase 1 (*ECHS1*), enoyl-CoA, hydratase/3-hydroxyacyl CoA dehydrogenase (*EHHADH*) and hydroxyacyl-CoA dehydrogenase (*HADH*), and 3 genes involved in fatty acid transportation such as solute carrier family 27 member 2 (*SLC27A2*), solute carrier family 27 member 4 (*SLC27A4*) and solute carrier family 27 member 5 (*SLC27A5*), were obviously upregulated in *SPOP*-mutated PCT tissues (Supplementary Figure 1A and Supplementary Figure 2). Additionally, 9 free fatty acids (FFAs) were found to increase significantly in *SPOP*-mutated PCT tissues in metabolomics profiling, including saturated and unsaturated fatty acids (Figure 3A–3I). These findings indicated that fatty acid metabolism might be a dominant energy source for tumor growth in *SPOP*-mutated PCa patients. Furthermore, integrated analysis was conducted by calculating Pearson correlation coefficients (PCC) between 58 transcripts and 52 metabolites involved in fatty acid metabolic pathways and showed strong correlations between transcripts and metabolites in *SPOP*-mutated cohort (Figure 3J). Of these transcripts, *ACADL* and *ELOVL2* exhibited the strongest correlations with most of fatty acid metabolites, suggesting that the alterations of *ACADL* and *ELOVL2* may be regulated by *SPOP* mutations.

In addition, glycerophospholipid metabolism was another remarkably altered pathway in *SPOP*-mutated cohort (Supplementary Figure 1B and Supplementary Table 1). Metabolomics and lipidomics analyses showed that most of the key intermediate metabolites involved in glycerophospholipid metabolism, such as PC and phosphatidylethanolamine (PE), were obviously increased in *SPOP*-mutated PCT tissues. Consistently, our results of transcriptomics also showed significant elevation of 8 involving genes, such as 1-acylglycerol-3-phosphate O-acyltransferase 6 (*AGPAT6*), membrane bound O-acyltransferase domain containing 2 (*MBOAT2*), lysocardiolipin acyltransferase 1 (*LCLAT1*), 1-acylglycerol-3-phosphate O-acyltransferase 3 (*AGPAT3*), ethanolaminephosphotransferase 1 (*EPT1*), lysophospholipase I (*LYPLA1*), phospholipase A2 group XIIA (*PLA2G12A*) and lysophosphatidylcholine acyltransferase 3 (*LPCAT3*) (Supplementary Figure 1B). These findings indicated that an overall upregulated glycerophospholipid metabolic pathway in *SPOP*-mutated PCT tissues.

**Figure 4:**
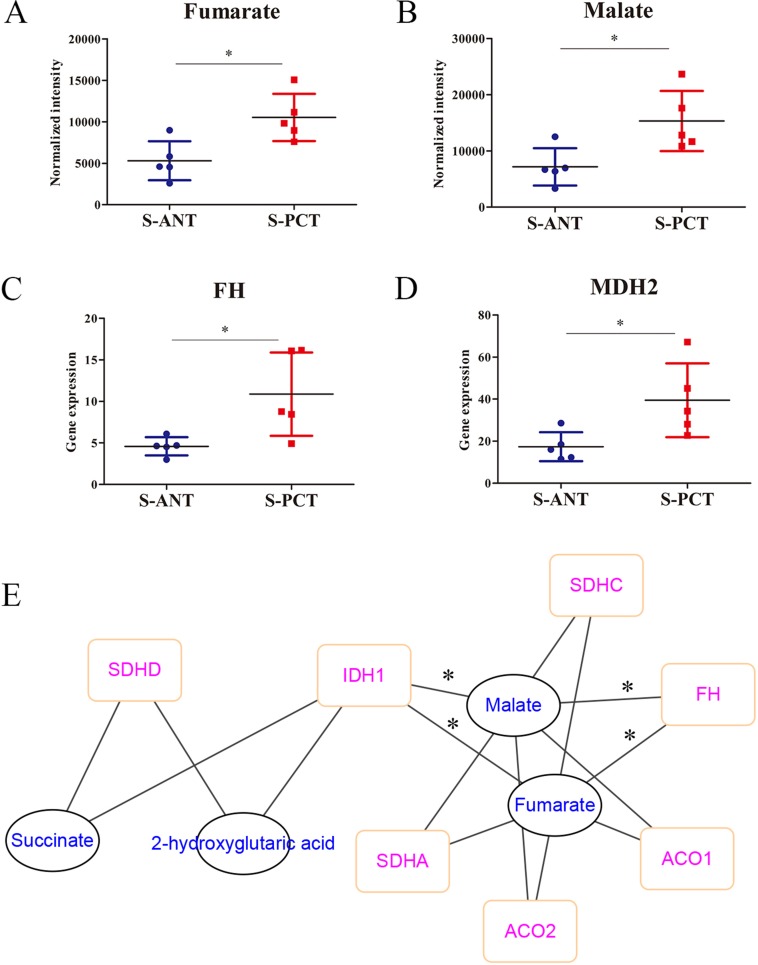
The alterations in TCA metabolic pathway in SPOP-mutated cohort (**A–B**) The normalized peak intensity values of fumarate (A) and malate (B). (**C**–**D**) The expression of *FH* (C) and *MDH2* (D) transcripts. (**E**) A correlation network was constructed based on PCC between transcripts and metabolites in TCA cycle, with the absolute value of correlation coefficient more than 0.6. ^*^p < 0.05 compared with the ANT tissues. S-PCT, *SPOP*-mutated PCT tissues; S-ANT, *SPOP*-mutated ANT tissues.

It is well-accepted that TCA cycle plays a critical role in cancer progression [29]. Here, based on the metabolomics analysis, fumarate and malate, two key intermediates involved in TCA cycle, were found to upregulate markedly in *SPOP*-mutated cohort (Figure 4A and 4B). Surprisingly, the catabolic enzymes of fumarate hydratase (*FH*) and malic dehydrogenase 2 (*MDH2*) transcripts were significantly upregulated in transcriptomics profiles in *SPOP*-mutated cohort (Figure 4C and 4D). Moreover, a correlation network based on PCC between transcripts and metabolites in TCA cycle showed obvious correlations between *FH*, isocitrate dehydrogenase 1 (*IDH1*) transcripts and fumarate as well as malate (Figure 4E). However, there was neither correlation between *MDH2* transcript and metabolites nor significant alteration for *IDH1* transcript in transcriptomics profiles. Hence, these alterations revealed that the obvious upregulation of *FH* gene in PCa might be associated with *SPOP* mutations.

### Transcriptomics and metabolomics differences between *SPOP*-WT (wild type) and *SPOP-*mutated PCT tissues

**Figure 5:**
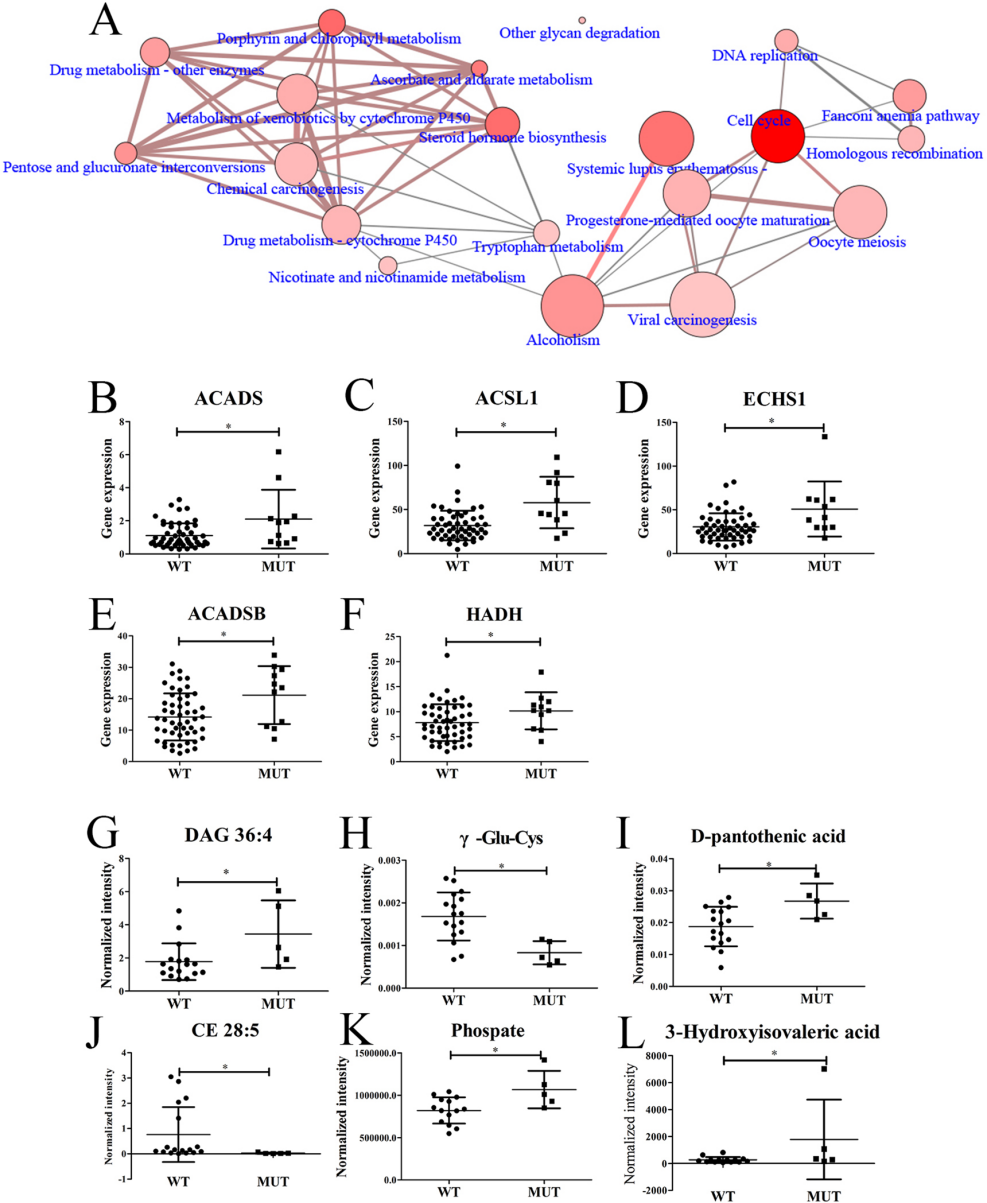
Differences between SPOP-mutated and SPOP-WT PCT tissues (**A**) Differential pathways between *SPOP*-mutated and *SPOP*-WT PCT tissues by transcriptomics analysis. (**B**–**F**) Differential fatty acid-related transcripts between *SPOP*-mutated and *SPOP*-WT PCT tissues, including (B) *ACADS,* (C) *ACSL1,* (D) *ECHS1,* (E) *ACADSB,* (F) *HADH.* (**G**–**L**) Differential metabolites between *SPOP*-mutated and *SPOP*-WT PCT tissues, including (G) DAG 36:4, (H) *γ-Glu-Cys,* (I) D-pantothenic acid, (J) CE 28:5, (K) Phosphate, (L) 3-Hydroxyisovaleric acid. ^*^ *p* < 0.05, *SPOP*-mutated compared with *SPOP*-WT PCT tissues. (A: the dot size indicated the gene number, the dot color indicated the *p* value, the edge width indicated the shared genes and the edge color indicated the genes form input. If the dot and edge color were deeper, smaller the *p* value or lager the genes form input was indicated. If the dot and edge was bigger or wider, lager gene numbers or shared genes were indicated. WT, *SPOP*-WT PCT tissues; MUT, *SPOP*-mutated PCT tissues).

To find out the transcriptomic differences between *SPOP*-WT and *SPOP*-mutated PCT tissues, the transcriptomic data from 11 *SPOP*-mutated PCT tissues were analyzed by comparing with those of 52 *SPOP*-WT PCT tissues. Totally, 1357 differential transcripts were found out and used to analyze the related pathways by Consensuspathdb [27]. Several pathways such as cell cycle, steroid hormone biosynthesis and others displayed obvious differences between *SPOP*-WT and *SPOP*-mutated PCT tissues (Figure 5A). Five transcripts related to fatty acid degradation, including acyl-CoA dehydrogenase, C-2 To C-3 short chain (*ACADS*), acyl-CoA synthetase long-chain family member 1 (*ACSL1*), *ECHS1*, *ACADSB* and *HADH*, were shown remarkable upregulation in *SPOP*-mutated PCT tissues (Figure 5B–5F), of which *ECHS1*, *ACADSB* and *HADH* were also upregulated significantly when compared to their matched ANT tissues. Moreover, based on the metabolomics and lipidomics data, the top six differential metabolites between *SPOP*-WT and *SPOP*-mutated PCT tissues were found, of which diacylglycerol (DAG) 36:4, as an important diacylglycerol metabolite, exhibited obvious increase in *SPOP*-mutated PCT tissue (Figure 5G–5L). These results were consistent with the alterations summarized in Supplementary Figure 1.

### Validation of the correlation between *SPOP* and metabolic genes including *FH*, *ELOVL2* and *ACADL*

To further validate the correctness of aforementioned findings, the big database of cBioportal [30, 31] was used to search for the alteration frequency of *SPOP*, *FH*, *ELOVL2* and *ACADL* genes in PCa and analyze the potential correlations of *SPOP* with *FH*, *ELOVL2* and *ACADL*. *ACTB* was used as negative control and *ERG* was used as positive control. Of note, *SPOP* (18%), *FH* (9%), *ELOVL2* (5%) and *ACADL* (5%) were significantly altered in PCa cohorts (Supplementary Figure 3A, 333 samples; Primary Prostate Carcinomas; TCGA, Cell 2015) [32]. Frequently, the mRNA expression of *FH*, *ELOVL2* and *ACADL* was upregulated in *SPOP* mutated patients (Supplementary Figure 3A). The *SPOP* mutations exhibited remarkable co-occurrence correlations with genetic alterations of *FH*, *ELOVL2* and *ACADL* and significant exclusivity correlation with *ERG*, but not with *ACTB* (Supplementary Figure 3B). These results were consistent with our findings of transcriptomics that the expression of *FH*, *ELOVL2* and *ACADL* were obviously upregulated in *SPOP* mutated patients.

**Figure 6:**
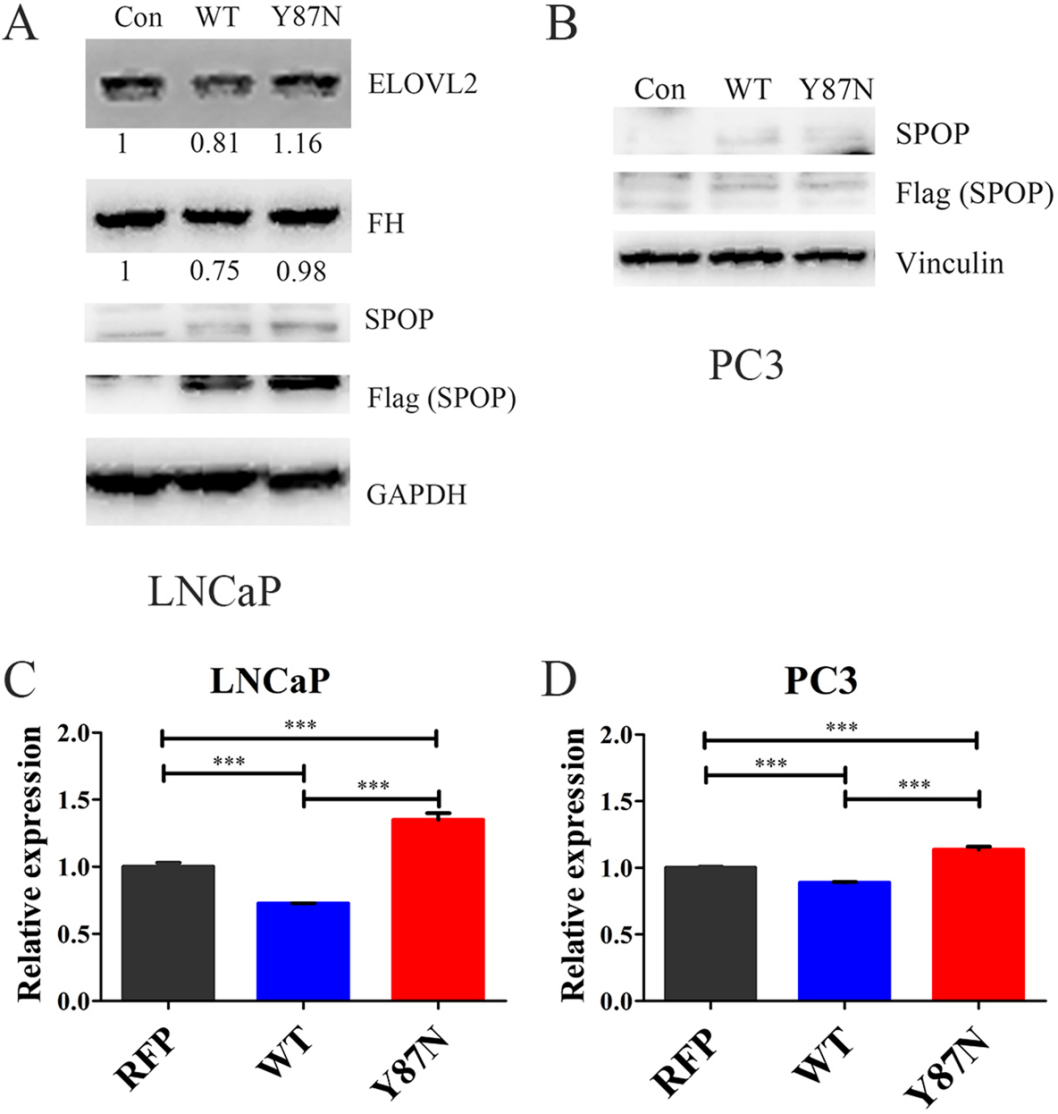
Expression of FH and ELOVL2 in SPOP_WT and SPOP_ Y87N transduced PCa cells (**A**–**B**) Immunoblotting of FH and ELOVL2 in SPOP_WT and SPOP_Y87N transduced LNCaP and PC3 cells. (**C**–**D**) QRT-PCR of *FH* in SPOP_WT and SPOP_Y87N transduced LNCaP and PC3 cells. *n* = 3 for each group. (Con, Control, PLOC.RFP vector; WT, SPOP_WT; Y87N, SPOP_Y87N.).

Furthermore, the expression of *FH*, *ELOVL2* and *ACADL* were validated in SPOP_WT and SPOP_Y87N transduced HEK293T, LNCaP and PC3 cells (Figure 6, Supplementary Figure 4). The protein levels of FH and ELOVL2 were significantly decreased in SPOP_WT transduced LNCaP cells compared with the control and SPOP_Y87N transduced LNCaP cells (Figure 6A). In 293T cells, transducing SPOP_WT caused a significant decrease in the protein levels of FH and ACADL compared with the control and SPOP_Y87N transduced cells (Supplementary Figure 4). Additionally, compared with the control as well as SPOP_Y87N transduced LNCaP and PC3 cells, the mRNA level of *FH* was also decreased in SPOP_WT transduced cells (Figure 6C–6D). Taken together, our findings suggest that *FH*, *ELOVL2* and *ACADL* genes might be the downstream transcripts of *SPOP* and play critical roles in the upregulation of metabolic pathways mediated by *SPOP* mutations.

## DISSCUSSION

*SPOP* is recognized as one of the most frequently mutated genes in PCa [5], which was also confirmed in our current study (Figure 1A). Increasing evidence supports the notion that *SPOP* mutations define a distinct molecular subclass of PCa [5, 9, 33]. Although a large body of studies focus on the functions of *SPOP* mutations in PCa, less is known about *SPOP*-mutation-related metabolic pathways. Here, our study reported for the first time that several altered metabolic pathways in PCT tissues might be intimately associated with *SPOP* mutations.

Traditionally, metabolomics and transcriptomics have been investigated alone. Transcriptomics, defined as a global expression profiling of RNA transcripts, has been widely applied as a valuable tool to comprehensively understand genetic alterations [34–36]. Metabolomics, as a global analysis of numerous metabolites, can provide data-rich information of metabolic alterations and is usually applied for discovering biomarkers [19, 37]. However, transcriptomics is limited to post-transcriptional modifications and metabolomics is limited to the functional analysis of metabolites. To avoid the limitations of single omics, integration of transcriptomics and metabolomics was performed in this study, which could precisely identify the altered metabolic pathways during disease progression and deeply understand the alterations of metabolites. Here, we investigated the alterations of metabolic pathways in *SPOP*-mutated PCa patients by integration of transcriptomics and metabolomics as well as lipidomics, followed by validation of the correlation between *SPOP* and metabolic genes through cBioportal. Importantly, our transcriptomics data and metabolomics data could support each other, which could also be confirmed by the results from correlation analysis, suggesting a high credibility.

It is well-known that *SPOP* usually acts as a tumor suppressor in PCa and somatic missense mutations of *SPOP* occur frequently in PCa [5]. AR has been identified as a central player in PCa progression including cell proliferation, migration and metabolism, and also a validated therapeutic target [38]. Several previous studies demonstrated that SPOP could directly bind to AR and contribute to its ubiquitination and degradation [9, 39]. Additionally, AR has been reported to be intimately associated with the alterations in metabolism and biosynthesis in PCa [40]. Hence, we speculated that there might be a relationship between *SPOP* mutations and metabolic alterations in PCa. As expected, numerous metabolites were found to express aberrantly in *SPOP*-mutated PCT tissues, most of which were significantly upregulated. Specifically, five of the top six remarkably upregulated metabolites were lipids (Figure 2B–2G). Moreover, almost all differential metabolites and transcripts involved in fatty acid metabolic pathway and glycerophospholipid metabolic pathway were notably upregulated in *SPOP*-mutated PCT tissues (Figure 3, Supplementary Figures 1–2 and Supplementary Table 1). Therefore, these findings suggest that lipid accumulation in PCT tissues may be closely associated with *SPOP* mutations.

TCA cycle plays a critical role in the transformation of energy metabolites and frequently dysregulated during cancer progression [29, 41]. In PCT tissues, TCA cycle is highly activated by loss of zinc accumulation, which can produce energy to fuel prostate tumorigenesis [42, 43]. In our data, fumarate and malate increased obviously in PCT tissues of *SPOP*-mutated cohort and exhibited highly correlations with the upregulation of *FH* by integration of transcriptomics and metabolomics (Figure 4). Surprisingly, it was also found that *SPOP* mutations positively correlated with upregulation of *FH* based on cBioportal analysis (Supplementary Figure 3). Our findings revealed that the increase of fumarate and malate in PCT tissues might be mediated by *SPOP* mutations at the transcriptional level (Supplementary Figure 3).

As another key metabolic pathway, fatty acid metabolism is usually upregulated in PCa [44], which is consistent with our results that various metabolites and transcripts were markedly upregulated in *SPOP*-mutated PCT tissues. Among these transcripts, *ACADL* has been identified as a key modulator in fatty acid β-oxidation and its expression can contribute to the malignant phenotypes of PCa cells [45]. Fatty acid oxidation is an important energy supplement in PCa [46]. Moreover, the over-expression of FASN, a key enzyme involved in fatty acid biosynthesis, can promote the PCa progression [47]. In the present study, *ACADL* showed notable upregulation and strong correlation with most of fatty acid metabolites in PCT tissues with *SPOP* mutations. In addition, *ELOVL2*, an elongase of long-chain fatty acid, also exhibited the similar alterations with *ACADL* in *SPOP*-mutated tissues (Figure 3J, Supplementary Figure 1A, and Supplementary Figure 2). Of note, cBioportal database showed a highly positive correlation between *SPOP* mutations and the genetic alteration of both two genes in primary PCa (Supplementary Figure 3). Therefore, our data indicated that the elevated fatty acid metabolism in PCa might be affected by *SPOP* mutations.

Although *FH*, *ELOVL2* and *ACADL* were recognized as key genes in *SPOP* mutated PCa patients in this study, their oncogenic roles still need to be proved in PCa. Previously, several studies have been reported that *FH* is frequently mutated in renal cancer [48], *ELOVL2* is upregulated in hepatocellular cancer [49], and *ACADL* is associated with prostate cancer progression[45]. Moreover, AR and ERG are important substrates of SPOP. AR, a type of nuclear receptor that is activated by binding to the androgenic hormones, can regulate eukaryotic gene expression and affect cellular proliferation and differentiation. Importantly, AR is highly activated in prostate cancer and fuels prostate cancer by upregulating glycolysis and fatty acid metabolism [23]. Also, AR transcriptional activity is increased in *SPOP* mutated prostate cancer [50]. In addition, ERG is an oncogenic regulator, which can modulate citrate, polyamines and choline-associated metabolites in PCa [51]. In this study, we found that fatty acid metabolism and choline-associated metabolism were upregulated in *SPOP* mutated PCa patients.

Moreover, the data of transcriptomics and metabolomics between *SPOP*-WT and *SPOP*-mutated PCT tissues were also analyzed and several critical metabolic pathways during PCa development showed obvious differences (Figure 5A). Among these pathways, steroid hormone pathway has been reported to be associated with the risk of PCa progression [52]. In addition, several important transcripts related to fatty acid metabolic pathway, especially *ECHS1*, *ACADSB* and *HADH*, were remarkably upregulated in *SPOP*-mutated PCT tissues when compared to their matched ANT tissues as well as *SPOP*-WT PCT tissues (Figure 5B–5F). Furthermore, DAG 36:4, as an important diacylglycerol metabolite, also obviously increased in *SPOP*-mutated PCT tissue (Figure 5G). Although lipid accumulation and upregulated fatty acid metabolism were found in PCT tissues in an overall analysis [21, 22], our findings demonstrated that such changes were more significant in *SPOP*-mutated PCT tissue. Nevertheless, no many differential metabolites between *SPOP*-WT and *SPOP*-mutated PCT tissues were found, which was probably due to the small size of samples. Hence, large-scale population-based studies are needed to be performed to further explore the *SPOP* mutation-related metabolic pathways as well as the molecular mechanisms.

In summary, various metabolic pathways were demonstrated upregulation at both transcriptional and metabolic levels in PCT tissues by integration of transcriptomics and metabolomics as well as lipidomics. Furthermore, we found that three upregulated metabolic pathways in PCT tissues including TCA cycle, fatty acid metabolism and glycerophospholipid metabolism, showed intimate association with *SPOP* mutations. Our study provided new insights in understanding the relationship between *SPOP* mutations and metabolic pathways in PCa. However, only the comparison of *SPOP* mutated PCT and ANT tissues is not enough to obtain the *SPOP* regulated metabolic pathways. Here, only a few differential metabolites were found between *SPOP* mutated and non-mutated PCT tissues. Additionally, *SPOP* mutated patients also obtained some other genetic alterations, which could be the main reason why only got few differences between *SPOP*-mutated and *SPOP-*WT cancer tissues. Further research would be needed to better understand such relationship in a large number of *SPOP*-mutated PCa patients as well as by functional detection *in vitro* and *in vivo*.

## MATERIALS AND METHODS

### Chemicals, reagents, plasmids and transfection

Ultrapure water was prepared using Milli-Q water purify system (Millipore, Billerica, MA, USA). Some chemical standards including methyl tert-butyl ether (MTBE), pyridine, dichloromethane, methoxyamine hydrochloride, (N-methyl-N-(trimethylsilyl)-trifluoroacetamide (MSTFA), ammonium acetate, formic acid and ammonium bicarbonate were obtained from Sigma-Aldrich (St. Louis, MO, USA), and other standards such as LC or MS grade methanol, acetonitrile, chloroform and isopropanol were purchased from Merck (Darmstodt, Germany).

*SPOP* WT plasmid was kindly provided by Prof. Jiang Liu from Beijing Institute of Genome Research, Chinese Academy of Sciences. *SPOP*-mutated plasmids Y87N were kindly provided by Dr. Chenji Wang from Fudan University. *SPOP* WT, Y87N were cloned into Ploc.RFP vector with 3 × Flag label. TurboFect Transfection Reagent (Thermo Scientific; Waltham, MA, USA) was used for transfection.

### Clinical sample collection and preparation

Totally, 66 patients were enrolled from Shanghai Changhai Hospital and wrote informed consent. The patient information of 66 PCa patients was listed on Table 1 with information of GS score, pathological stage and pathological progression, including *SPOP*-WT and *SPOP*-mutated PCa patients. All experimental protocols were approved by Institutional Review Board of the Shanghai Changhai Hospital, Second Military Medical University, Shanghai, China. All matched ANT and PCT tissues were obtained from surgery, flash-frozen in liquid nitrogen and kept at –80°C until analysis. Hematoxylin and eosin staining was used for histological diagnosis. In *SPOP*-WT and *SPOP*-mutated patient comparison, the *SPOP*-WT and *SPOP*-mutated patients were analyzed at the same batch by metabolomics and lipidomics studies and the patient information had been listed with *SPOP*-WT and *SPOP*-mutated PCa patients [21].

### LC-MS based metabolomics and lipidomics analyses

LC-MS based metabolomics and lipidomics analyses of PCT and ANT tissues were performed as previously described [20, 21]. Briefly, the extraction of metabolites was performed using a system containing menthol, MTBE and water. ACQUITY^™^ Ultra Performance Liquid Chromatography (UPLC) system (Waters, Milford, MA, USA) was used for chromatographic separation, followed by global metabolomics and lipidomics profiling performed by a coupled AB Sciex tripleTOF 5600 plus mass spectrometer (Applied Biosystems Sciex, Foster City, CA, USA).

### GC-MS based metabolomics analysis

GC-MS based metabolic profiling of PCT and ANT tissues was analyzed using GCMS-QP 2010 analytical system (Shimadzu, Kyoto, Japan) equipped with EI (electron impact) ionization source as previously described with slight modifications [53]. In brief, the extraction of metabolites was conducted using a system containing 80% menthol, water and 10 μg/mL tridecanoic acid on ice. Chromatographic separation of metabolites was performed on a DB-5 MS capillary column (J & W scientific, Folsom, CA, USA).

### Transcriptomics analysis

The data of gene expression and somatic mutations were obtained using RNA-seq analysis as previously described [54]. Briefly, the total RNA was extracted by using TRIzol reagent and cDNA sequencing was performed by using Illumina Kit (San Diego, CA, USA) according to the manufacturers’ instructions. TopHat software was used to calculate the clean reason of RNA nucleotide sequences.

### cBioportal data analysis

Genetic alteration frequency of *SPOP*, *ACADL*, *ELOVL2*, *FH*, *ACTB* and *ERG* in PCa was analyzed using cBioportal (http://www.cbioportal.org/index.do) [30, 31]. All searches were conducted according to the online protocols of cBioportal. Additionally, the correlation analyses between *SPOP* and *FH*, *ELOVL2* as well as *ACADL* were performed based on their mutation and expression in primary prostate carcinomas (TCGA, Cell 2015).

### Immunoblotting

Immunoblotting was performed as previously described [21]. Cells were prepared by RIPA (radio-immunoprecipitation assay) buffer with phosphatase inhibitors (Sigma). After vortex 30 s, cells were lysed at ice for 15 min and centrifuged at 12000 rpm for 15 min at 4°C. Supernatant was denatured and used for sodium dodecyl sulphate-polyacrylamide gel electrophoresis (SDS-PAGE) separation. The proteins were then transferred to a polyvinylidene fluoride (PVDF) membrane (Bio-Rad; Hercules, CA, USA). Subsequently, the membranes were immunoblotted with the corresponding primary antibodies followed by peroxidase-conjugated secondary antibodies. The bonds were visualized by Chemiluminescence (Thermo Scientific; Waltham, MA, USA). SPOP (1:1000 Proteintech; 16750-1-AP), Flag (1:1000 Proteintech; 66008-2-Ig), GAPDH (1:2000 Cell Signaling Technology; #5174), FH (1:1000 Proteintech; 10966-1-AP), ELOVL2 (1:500 Abcam; EPR11880), ACADL (1:1000 Proteintech; 17526-1-AP) and Vinculin (1:2000 Sigma; V4505) antibodies were used in the study.

### Real-time PCR

For real-time PCR analysis, mRNA was extracted by RNAiso Plus (Takara, Dalian, China). Then, the mRNA was reverse transcription to cDNA by PrimeScript™ RT reagent Kit with gDNA Eraser (Perfect Real Time) (Takara, Dalian, China). SYBR^®^ Premix Ex Taq™ (Tli RNaseH Plus) (Takara, Dalian, China) was used for quantitative analysis. The CFX96™ Real-Time PCR Detection Systems was used. The FH primers were: FH-F, GGAGGTGTGACAGAACGCAT; FH-R, CATCTGCTGCCTTCATTATTGC; ACTB-F; TGACGTGGACATCCGCAAAG; ACTB-R, CTGGAAGGTGGACAGCGAGG.

### Data processing and statistics

For comparing the differential metabolites between the matched PCT and ANT tissues in *SPOP*-mutated patients in metabolomics and lipidomics profiling, two-sided Mann–Whitney U test was used and *P* < 0.05 was considered to indicate statistical significance. The heatmap was visualized using Multi Experiment Viewer (MeV, version 4.8.1) according to the ratios of normalized peak intensity between paired PCT and ANT tissues [55]. Two-sided Mann–Whitney U test (Matlab, 2014b) was also used to find out the differential transcripts between 11 matched PCT and ANT tissues in *SPOP*-mutated patients. *P*-value was set less than 0.05 and fold change was set more than 2 or less than 0.5. A total of 1840 upregulated and 1926 down-regulated transcripts were used for gene set analysis by ConsensusPathDB (http://consensuspathdb.org/) [27], and KEGG [26] was used as database. Minimum overlap with input list was defined as 2 and *P* < 0.01 was set as statistically significant. The comparison of *SPOP* mutated and not mutated PCT tissues were calculated by two-sided Mann–Whitney U test and *P* < 0.05 was considered statistical significance.

The ratios of metabolites and transcripts in PCT tissues to that in matched ANT tissues were calculated using normalized peak intensity of metabolites and gene expression data, respectively. The correlations between PCT to ANT ratio of transcripts and metabolites were analyzed based on PCC (Matlab, 2014b) and the correlation network was visualized using cytoscape (3.3.0) [56]. Human metabolome database (HMDB) was used to search for the functions of metabolites (http://www.hmdb.ca/) [57]. The networks between metabolites and genes were searched at KEGG (http://www.kegg.jp/kegg/) [26]. The Scatter plot is drawn by GraphPad Prism 5.

## SUPPLEMENTARY MATERIALS FIGURES AND TABLE



## References

[1] Khazaei S, Rezaeian S, Ayubi E, Gholamaliee B, Pishkuhi MA, Khazaei S, Mansori K, Nematollahi S, Sani M, Hanis SM (2016). Global Prostate Cancer Incidence and Mortality Rates According to the Human Development Index. Asian Pac J Cancer Prev.

[2] Wong MC, Goggins WB, Wang HH, Fung FD, Leung C, Wong SY, Ng CF, Sung JJ (2016). Global Incidence and Mortality for Prostate Cancer: Analysis of Temporal Patterns and Trends in 36 Countries. Eur Urol.

[3] Chen W, Zheng R, Baade PD, Zhang S, Zeng H, Bray F, Jemal A, Yu XQ, He J (2016). Cancer statistics in China, 2015. CA Cancer J Clin.

[4] Center MM, Jemal A, Lortet-Tieulent J, Ward E, Ferlay J, Brawley O, Bray F (2012). International variation in prostate cancer incidence and mortality rates. Eur Urol.

[5] Barbieri CE, Baca SC, Lawrence MS, Demichelis F, Blattner M, Theurillat JP, White TA, Stojanov P, Van Allen E, Stransky N, Nickerson E, Chae SS, Boysen G (2012). Exome sequencing identifies recurrent SPOP, FOXA1 and MED12 mutations in prostate cancer. Nat Genet.

[6] Zhuang M, Calabrese MF, Liu J, Waddell MB, Nourse A, Hammel M, Miller DJ, Walden H, Duda DM, Seyedin SN, Hoggard T, Harper JW, White KP, Schulman BA (2009). Structures of SPOP-substrate complexes: insights into molecular architectures of BTB-Cul3 ubiquitin ligases. Mol Cell.

[7] Theurillat JP, Udeshi ND, Errington WJ, Svinkina T, Baca SC, Pop M, Wild PJ, Blattner M, Groner AC, Rubin MA, Moch H, Prive GG, Carr SA, Garraway LA (2014). Prostate cancer. Ubiquitylome analysis identifies dysregulation of effector substrates in SPOP-mutant prostate cancer. Science.

[8] Geng C, He B, Xu L, Barbieri CE, Eedunuri VK, Chew SA, Zimmermann M, Bond R, Shou J, Li C, Blattner M, Lonard DM, Demichelis F (2013). Prostate cancer-associated mutations in speckle-type POZ protein (SPOP) regulate steroid receptor coactivator 3 protein turnover. Proc Natl Acad Sci USA.

[9] An J, Wang C, Deng Y, Yu L, Huang H (2014). Destruction of full-length androgen receptor by wild-type SPOP, but not prostate-cancer-associated mutants. Cell Reports.

[10] Gan W, Dai X, Lunardi A, Li Z, Inuzuka H, Liu P, Varmeh S, Zhang J, Cheng L, Sun Y, Asara JM, Beck AH, Huang J (2015). SPOP Promotes Ubiquitination and Degradation of the ERG Oncoprotein to Suppress Prostate Cancer Progression. Mol Cell.

[11] An J, Ren S, Murphy SJ, Dalangood S, Chang C, Pang X, Cui Y, Wang L, Pan Y, Zhang X, Zhu Y, Wang C, Halling GC (2015). Truncated ERG Oncoproteins from TMPRSS2-ERG Fusions Are Resistant to SPOP-Mediated Proteasome Degradation. Mol Cell.

[12] Wu F, Dai X, Gan W, Wan L, Li M, Mitsiades N, Wei W, Ding Q, Zhang J (2017). Prostate cancer-associated mutation in SPOP impairs its ability to target Cdc20 for poly-ubiquitination and degradation. Cancer Lett.

[13] Dang L, White DW, Gross S, Bennett BD, Bittinger MA, Driggers EM, Fantin VR, Jang HG, Jin S, Keenan MC, Marks KM, Prins RM, Ward PS (2009). Cancer-associated IDH1 mutations produce 2-hydroxyglutarate. Nature.

[14] Boroughs LK, DeBerardinis RJ (2015). Metabolic pathways promoting cancer cell survival and growth. Nat Cell Biol.

[15] Huang Q, Tan Y, Yin P, Ye G, Gao P, Lu X, Wang H, Xu G (2013). Metabolic characterization of hepatocellular carcinoma using nontargeted tissue metabolomics. Cancer Res.

[16] Wu X, Deng F, Li Y, Daniels G, Du X, Ren Q, Wang J, Wang LH, Yang Y, Zhang V, Zhang D, Ye F, Melamed J (2015). ACSL4 promotes prostate cancer growth, invasion and hormonal resistance. Oncotarget.

[17] Warburg O (1956). On the origin of cancer cells. Science.

[18] McDunn JE, Li Z, Adam KP, Neri BP, Wolfert RL, Milburn MV, Lotan Y, Wheeler TM (2013). Metabolomic signatures of aggressive prostate cancer. Prostate.

[19] Sreekumar A, Poisson LM, Rajendiran TM, Khan AP, Cao Q, Yu J, Laxman B, Mehra R, Lonigro RJ, Li Y, Nyati MK, Ahsan A, Kalyana-Sundaram S (2009). Metabolomic profiles delineate potential role for sarcosine in prostate cancer progression. Nature.

[20] Ren S, Shao Y, Zhao X, Hong CS, Wang F, Lu X, Li J, Ye G, Yan M, Zhuang Z, Xu C, Xu G, Sun Y (2016). Integration of Metabolomics and Transcriptomics Reveals Major Metabolic Pathways and Potential Biomarker Involved in Prostate Cancer. Mol Cell Proteomics.

[21] Li J, Ren S, Piao HL, Wang F, Yin P, Xu C, Lu X, Ye G, Shao Y, Yan M, Zhao X, Sun Y, Xu G (2016). Integration of lipidomics and transcriptomics unravels aberrant lipid metabolism and defines cholesteryl oleate as potential biomarker of prostate cancer. Sci Rep.

[22] Liu Y (2006). Fatty acid oxidation is a dominant bioenergetic pathway in prostate cancer. Prostate Cancer Prostatic Dis.

[23] Massie CE, Lynch A, Ramos-Montoya A, Boren J, Stark R, Fazli L, Warren A, Scott H, Madhu B, Sharma N, Bon H, Zecchini V, Smith DM (2011). The androgen receptor fuels prostate cancer by regulating central metabolism and biosynthesis. EMBO J.

[24] Dasgupta S, Putluri N, Long W, Zhang B, Wang J, Kaushik AK, Arnold JM, Bhowmik SK, Stashi E, Brennan CA, Rajapakshe K, Coarfa C, Mitsiades N (2015). Coactivator SRC-2-dependent metabolic reprogramming mediates prostate cancer survival and metastasis. J Clin Invest.

[25] Priolo C, Loda M (2015). Untargeted metabolomics for profiling oncogene-specific metabolic signatures of prostate cancer. Mol Cell Oncol.

[26] Kanehisa M, Goto S, Sato Y, Kawashima M, Furumichi M, Tanabe M (2014). Data, information, knowledge and principle: back to metabolism in KEGG. Nucleic Acids Res.

[27] Kamburov A, Stelzl U, Lehrach H, Herwig R (2013). The ConsensusPathDB interaction database: 2013 update. Nucleic Acids Res.

[28] Huang M, Koizumi A, Narita S, Inoue T, Tsuchiya N, Nakanishi H, Numakura K, Tsuruta H, Saito M, Satoh S, Nanjo H, Sasaki T, Habuchi T (2016). Diet-induced alteration of fatty acid synthase in prostate cancer progression. Oncogenesis.

[29] Desideri E, Vegliante R, Ciriolo MR (2015). Mitochondrial dysfunctions in cancer: genetic defects and oncogenic signaling impinging on TCA cycle activity. Cancer Lett.

[30] Cerami E, Gao J, Dogrusoz U, Gross BE, Sumer SO, Aksoy BA, Jacobsen A, Byrne CJ, Heuer ML, Larsson E, Antipin Y, Reva B, Goldberg AP (2012). The cBio cancer genomics portal: an open platform for exploring multidimensional cancer genomics data. Cancer Discov.

[31] Gao J, Aksoy BA, Dogrusoz U, Dresdner G, Gross B, Sumer SO, Sun Y, Jacobsen A, Sinha R, Larsson E, Cerami E, Sander C, Schultz N (2013). Integrative analysis of complex cancer genomics and clinical profiles using the cBioPortal. Sci Signal.

[32] Abeshouse A, Ahn J, Akbani R, Ally A, Amin S, Andry Christopher D, Annala M, Aprikian A, Armenia J, Arora A, Auman JT, Balasundaram M, Balu S, Cancer Genome Atlas Research Network (2015). The Molecular Taxonomy of Primary Prostate Cancer. Cell.

[33] Blattner M, Lee DJ, O’Reilly C, Park K, MacDonald TY, Khani F, Turner KR, Chiu YL, Wild PJ, Dolgalev I, Heguy A, Sboner A, Ramazangolu S (2014). SPOP mutations in prostate cancer across demographically diverse patient cohorts. Neoplasia.

[34] Wang Z, Gerstein M, Snyder M (2009). RNA-Seq: a revolutionary tool for transcriptomics. Nat Rev Genet.

[35] Stephens NA, Gallagher IJ, Rooyackers O, Skipworth RJ, Tan BH, Marstrand T, Ross JA, Guttridge DC, Lundell L, Fearon KC, Timmons JA (2010). Using transcriptomics to identify and validate novel biomarkers of human skeletal muscle cancer cachexia. Genome Med.

[36] Bochmann L, Sarathchandra P, Mori F, Lara-Pezzi E, Lazzaro D, Rosenthal N (2010). Revealing new mouse epicardial cell markers through transcriptomics. PLoS One.

[37] Chen J, Zhang X, Cao R, Lu X, Zhao S, Fekete A, Huang Q, Schmitt-Kopplin P, Wang Y, Xu Z, Wan X, Wu X, Zhao N (2011). Serum 27-nor-5β-cholestane-3,7,12,24,25 pentol glucuronide discovered by metabolomics as potential diagnostic biomarker for epithelium ovarian cancer. J Proteome Res.

[38] Mitsiades N (2013). A road map to comprehensive androgen receptor axis targeting for castration-resistant prostate cancer. Cancer Res.

[39] Geng C, Rajapakshe K, Shah SS, Shou J, Eedunuri VK, Foley C, Fiskus W, Rajendran M, Chew SA, Zimmermann M, Bond R, He B, Coarfa C, Mitsiades N (2014). Androgen receptor is the key transcriptional mediator of the tumor suppressor SPOP in prostate cancer. Cancer Res.

[40] Barfeld SJ, Itkonen HM, Urbanucci A, Mills IG (2014). Androgen-regulated metabolism and biosynthesis in prostate cancer. Endocr Relat Cancer.

[41] Chen JQ, Russo J (2012). Dysregulation of glucose transport, glycolysis, TCA cycle and glutaminolysis by oncogenes and tumor suppressors in cancer cells. Biochim Biophys Acta.

[42] Costello LC, Franklin RB (1998). Novel role of zinc in the regulation of prostate citrate metabolism and its implications in prostate cancer. Prostate.

[43] Dakubo GD, Parr RL, Costello LC, Franklin RB, Thayer RE (2006). Altered metabolism and mitochondrial genome in prostate cancer. J Clin Pathol.

[44] Currie E, Schulze A, Zechner R, Walther TC, Farese RV (2013). Cellular fatty acid metabolism and cancer. Cell Metab.

[45] Xie BX, Zhang H, Wang J, Pang B, Wu RQ, Qian XL, Yu L, Li SH, Shi QG, Huang CF, Zhou JG (2011). Analysis of differentially expressed genes in LNCaP prostate cancer progression model. J Androl.

[46] Itkonen HM, Brown M, Urbanucci A, Tredwell G, Ho Lau C, Barfeld S, Hart C, Guldvik IJ, Takhar M, Heemers HV, Erho N, Bloch K, Davicioni E (2017). Lipid degradation promotes prostate cancer cell survival. Oncotarget.

[47] Menendez JA, Lupu R (2007). Fatty acid synthase and the lipogenic phenotype in cancer pathogenesis. Nat Rev Cancer.

[48] Isaacs JS, Jung YJ, Mole DR, Lee S, Torres-Cabala C, Chung YL, Merino M, Trepel J, Zbar B, Toro J, Ratcliffe PJ, Linehan WM, Neckers L (2005). HIF overexpression correlates with biallelic loss of fumarate hydratase in renal cancer: novel role of fumarate in regulation of HIF stability. Cancer Cell.

[49] Zekri AR, Hassan ZK, Bahnassy AA, Sherif GM, ELdahshan D, Abouelhoda M, Ali A, Hafez MM (2012). Molecular prognostic profile of Egyptian HCC cases infected with hepatitis C virus. Asian Pac J Cancer Prev.

[50] Cancer Genome Atlas Research N (2015). The Molecular Taxonomy of Primary Prostate Cancer. Cell.

[51] Hansen AF, Sandsmark E, Rye MB, Wright AJ, Bertilsson H, Richardsen E, Viset T, Bofin AM, Angelsen A, Selnæs KM, Bathen TF, Tessem MB (2016). Presence of TMPRSS2-ERG is associated with alterations of the metabolic profile in human prostate cancer. Oncotarget.

[52] Belledant A, Hovington H, Garcia L, Caron P, Brisson H, Villeneuve L, Simonyan D, Têtu B, Fradet Y, Lacombe L, Guillemette C, Lévesque E (2016). The UGT2B28 Sex-steroid Inactivation Pathway Is a Regulator of Steroidogenesis and Modifies the Risk of Prostate Cancer Progression. Eur Urol.

[53] Ye G, Liu Y, Yin P, Zeng Z, Huang Q, Kong H, Lu X, Zhong L, Zhang Z, Xu G (2014). Study of induction chemotherapy efficacy in oral squamous cell carcinoma using pseudotargeted metabolomics. J Proteome Res.

[54] Ren S, Peng Z, Mao JH, Yu Y, Yin C, Gao X, Cui Z, Zhang J, Yi K, Xu W, Chen C, Wang F, Guo X (2012). RNA-seq analysis of prostate cancer in the Chinese population identifies recurrent gene fusions, cancer-associated long noncoding RNAs and aberrant alternative splicings. Cell Res.

[55] Saeed AI, Sharov V, White J, Li J, Liang W, Bhagabati N, Braisted J, Klapa M, Currier T, Thiagarajan M, Sturn A, Snuffin M, Rezantsev A (2003). TM4: a free, open-source system for microarray data management and analysis. Biotechniques.

[56] Cline MS, Smoot M, Cerami E, Kuchinsky A, Landys N, Workman C, Christmas R, Avila-Campilo I, Creech M, Gross B, Hanspers K, Isserlin R, Kelley R (2007). Integration of biological networks and gene expression data using Cytoscape. Nat Protoc.

[57] Wishart DS, Jewison T, Guo AC, Wilson M, Knox C, Liu Y, Djoumbou Y, Mandal R, Aziat F, Dong E, Bouatra S, Sinelnikov I, Arndt D (2013). HMDB 3.0—The Human Metabolome Database in 2013. Nucleic Acids Res.

