# Supplementary material for: Identification of SPOP related metabolic pathways in prostate cancer
Source: Oncotarget. 2017 Oct 3;8(61):103032–46. doi: 10.18632/oncotarget.21460 (PMC5732708; doi:10.18632/oncotarget.21460)
Supplement: Supplementary file 1 [file oncotarget-08-103032-s001.pdf]

Identification of *SPOP* related metabolic pathways in prostate cancer

SUPPLEMENTARY MATERIALS

Supplementary Table 1: Differential metabolites were listed with *P* values, fold changes and metabolic pathways in *SPOP*-mutated cohort

Metabolites	*P* value	Fold change (T/N)	Platform	Pathways
N-Acetyl-D-mannosamine	0.03	2.68	GC-MS	Amino sugar and nucleotide sugar metabolism
N-Acetyl-D-Glucosamine 6-Phosphate	0.03	2.53	LC-MS	Amino sugar and nucleotide sugar metabolism
Carnitine C8:0	0.01	0.32	LC-MS	Beta Oxidation of Very Long Chain Fatty Acids
GCDCA	0.01	0.55	LC-MS	Bile acid biosynthesis
Nε,Nε,Nε-Trimethyl-lysine	0.03	2.53	LC-MS	Carnitine Synthesis
FFA 20:2	0.03	3.32	LC-MS	Fatty acid metabolism
FFA 22:2	0.02	2.86	LC-MS	Fatty acid metabolism
FFA 20:1	0.03	2.50	LC-MS	Fatty acid metabolism
FFA 20:3	0.03	2.42	LC-MS	Fatty acid metabolism
FFA 22:3	0.01	2.39	LC-MS	Fatty acid metabolism
FFA 22:0	0.02	2.05	LC-MS	Fatty acid metabolism
FFA 19:0	0.03	1.91	LC-MS	Fatty acid metabolism
FFA 20:0	0.03	1.89	LC-MS	Fatty acid metabolism
Cis-5,8,11-Eicosatrienoic acid	0.03	1.83	GC-MS	Fatty acid metabolism
FFA 22:4	0.03	1.79	LC-MS	Fatty acid metabolism
Oleic acid	0.03	1.60	GC-MS	Fatty acid metabolism
Hexadecanoic acid	0.03	1.54	GC-MS	Fatty acid metabolism
Pyroglutamic acid	0.03	1.57	GC-MS	Glutathione metabolism
TAG 55:1	0.03	6.09	Lipidomics	Glycerolipid metabolism
TAG 56:1	0.03	4.03	Lipidomics	Glycerolipid metabolism
TAG 58:1	0.03	3.54	Lipidomics	Glycerolipid metabolism
TAG 54:0	0.02	3.11	Lipidomics	Glycerolipid metabolism
TAG 52:0	0.02	3.02	Lipidomics	Glycerolipid metabolism
TAG 51:0	0.03	2.74	Lipidomics	Glycerolipid metabolism
TAG 56:0	0.02	2.38	Lipidomics	Glycerolipid metabolism
TAG 50:0	0.03	2.15	Lipidomics	Glycerolipid metabolism
PC 28:0	0.03	4.42	LC-MS	Glycerophospholipid metabolism
Choline glycerophosphate	0.03	4.37	LC-MS	Glycerophospholipid metabolism
PE O-36:4	0.03	4.24	Lipidomics	Glycerophospholipid metabolism
Glycerylphosphorylethanolamine	0.03	4.13	LC-MS	Glycerophospholipid metabolism
PC 37:1	0.03	3.66	Lipidomics	Glycerophospholipid metabolism
PC 35:1	0.03	3.51	Lipidomics	Glycerophospholipid metabolism
PC 33:1	0.03	2.38	Lipidomics	Glycerophospholipid metabolism
PE 35:1	0.02	2.19	Lipidomics	Glycerophospholipid metabolism
PE 33:1	0.03	1.86	Lipidomics	Glycerophospholipid metabolism
DAG 38:2	0.02	3.59	Lipidomics	Glycerophospholipid metabolism, Glycerolipid metabolism
DAG 36:3	0.02	2.11	Lipidomics	Glycerophospholipid metabolism, Glycerolipid metabolism
DAG 36:2	0.03	1.96	Lipidomics	Glycerophospholipid metabolism, Glycerolipid metabolism
DAG 34:2	0.01	1.71	Lipidomics	Glycerophospholipid metabolism, Glycerolipid metabolism
Histamine	0.03	0.44	LC-MS	Histidine metabolism
Glycerol-2-phosphate	0.03	2.23	GC-MS	Lipids metabolism
Glycerol 3-phosphate	0.03	2.02	LC-MS	Lipids metabolism
Arabinitol	0.01	1.86	GC-MS	Pentose and glucuronate interconversions
Cer 38:2;2	0.03	6.37	Lipidomics	Sphingolipid metabolism
Cer 34:2;2	0.02	3.58	Lipidomics	Sphingolipid metabolism
Cer 40:2;2	0.02	3.51	Lipidomics	Sphingolipid metabolism
CE 24:5	0.02	21.79	Lipidomics	Steroid biosynthesis
CE 20:1	0.03	11.02	Lipidomics	Steroid biosynthesis
Fumaric acid	0.02	2.51	LC-MS	TCA cycle
Malic acid	0.03	2.14	GC-MS	TCA cycle
Nicotinamide adenine dinucleotide	0.02	27.38	LC-MS	TCA cycle, Glycolysis

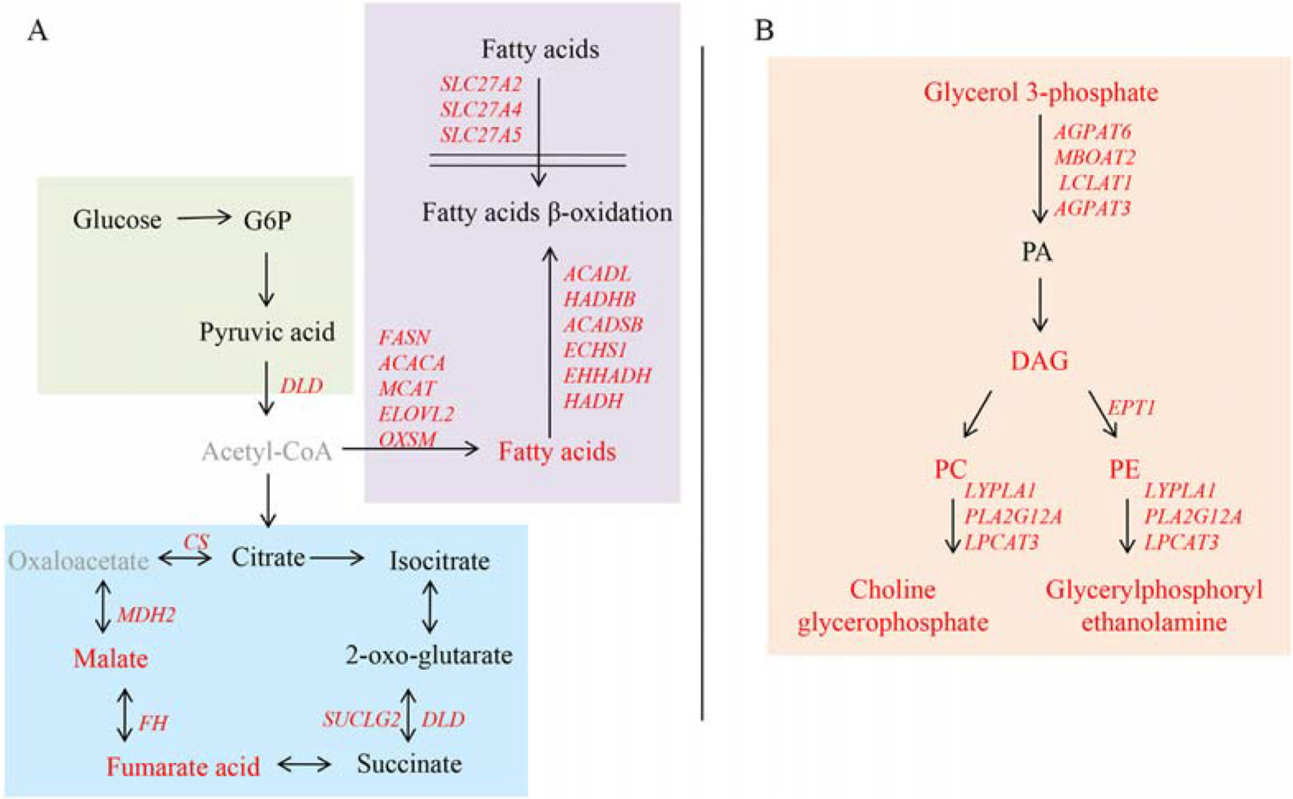

Supplementary Figure 1: Overall alterations of metabolic pathways in *SPOP*-mutated PCa patients. (**A**) Glycolysis metabolic pathway, TCA cycle pathway and fatty acids metabolic pathway are presented. (**B**) Glycerophospholipid metabolic pathway is shown. Black, Red, and grey fonts represent non-altered, significantly upregulated and non-detected metabolites and transcripts, respectively. The transcripts are labeled by *Italic* fonts.

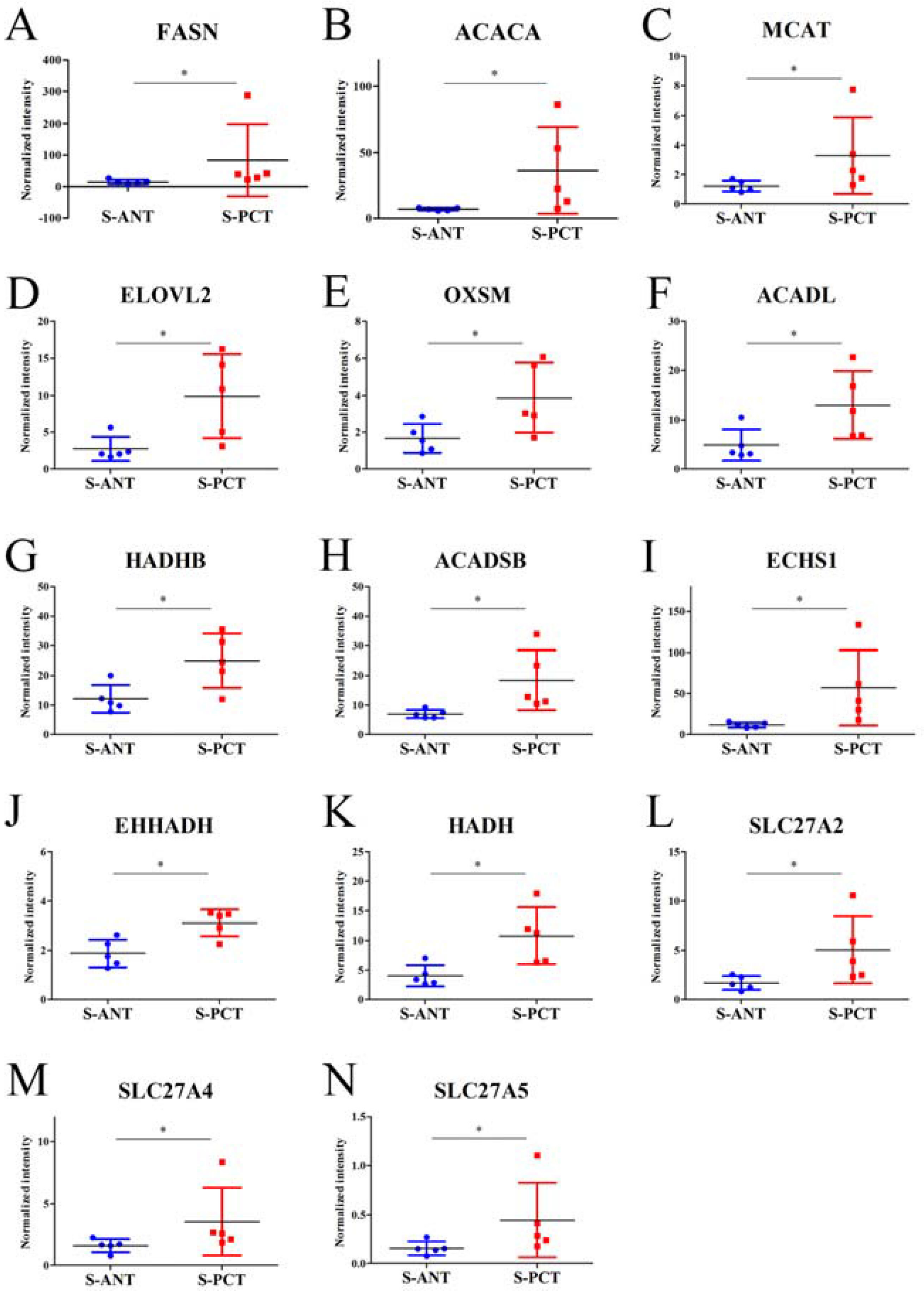

Supplementary Figure 2: Differentially expressed transcripts between the matched PCT and ANT tissues in fatty acid metabolic pathway in *SPOP*-mutated cohort. (**A**) *FASN*, (**B**) *ACACA*, (**C**) *MCAT*, (**D**) *ELOVL2*, (**E**) *OXSM*, (**F**) *ACADL*, (**G**) *HADHB*, (**H**) *ACADSB*, (**I**) *ECHS1*, (**J**) *EHHADH*, (**K**) *HADH*, (**L**) *SLC27A2*, (**M**) *SLC27A4* and (**N**) *SLC27A5* are shown. * $p < 0.05$ compared with the ANT tissues. S-PCT, *SPOP*-mutated PCT tissues; S-ANT, *SPOP*-mutated ANT tissues.

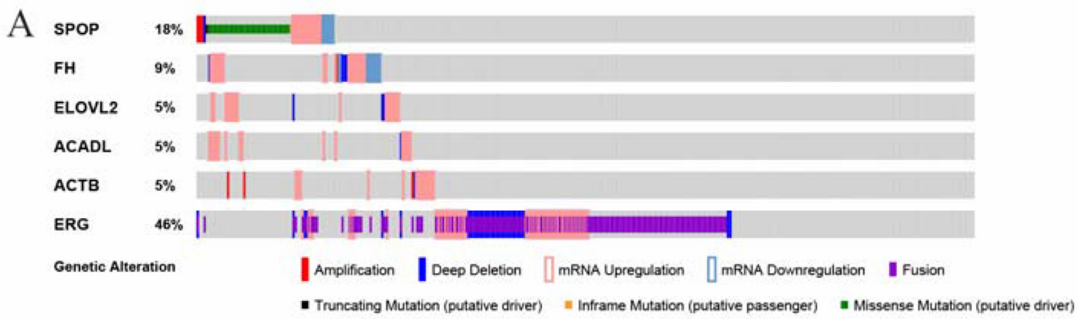

B

Gene A	Gene B	p-Value	Log Odds Ratio	Association
SPOP	*FH*	0.05	0.83	Tendency towards co-occurrence (Significant)
SPOP	*ELOVL2*	<0.01	1.67	Tendency towards co-occurrence (Significant)
SPOP	*ACADL*	<0.01	2.08	Tendency towards co-occurrence (Significant)
SPOP	*ACTB*	0.16	0.70	Tendency towards co-occurrence
SPOP	*ERG*	<0.01	-1.56	Tendency towards mutual exclusivity (Significant)

Supplementary Figure 3: The correlations of *SPOP* with *FH, ELOVL2, ACADL, ACTB* and *ERG* in PCa. (**A**) Genetic alterations of *SPOP, FH, ELOVL2, ACADL, ACTB* and *ERG* genes in PCa patients. (**B**) The correlations of SPOP with *FH, ELOVL2, ACADL, ACTB* and *ERG* in PCa were analyzed by cBioportal.

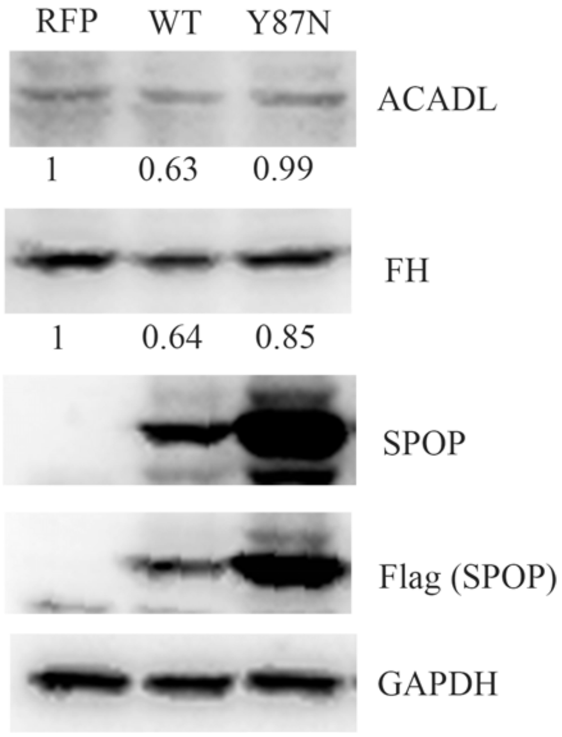

Supplementary Figure 4: Immunoblotting of ACADL and FH in SPOP_WT and SPOP_Y87N transduced HEK293T cells. (Con, Control, PLOC.RFP vector; WT, SPOP_WT; Y87N, SPOP_Y87N.).
